# Computational Discovery of Marine Molecules of the Cyclopeptide Family with Therapeutic Potential

**DOI:** 10.3390/ph16101377

**Published:** 2023-09-28

**Authors:** Norma Flores-Holguín, Joan S. Salas-Leiva, Daniel Glossman-Mitnik

**Affiliations:** Departamento de Medio Ambiente y Energía, Centro de Investigación en Materiales Avanzados, Miguel de Cervantes 120, Complejo Industrial Chihuahua, Chihuahua 31136, Mexico; norma.flores@cimav.edu.mx (N.F.-H.); joan.salas@cimav.edu.mx (J.S.S.-L.)

**Keywords:** drug discovery, computational chemistry, conceptual DFT, marine cyclopeptides, chemical reactivity properties, bioactivity scores, biological targets

## Abstract

Stellatolides are natural compounds that have shown promising biological activities, including antitumor, antimicrobial, and anti-inflammatory properties, making them potential candidates for drug development. Chemical Reactivity Theory (CRT) is a branch of chemistry that explains and predicts the behavior of chemical reactions based on the electronic structure of molecules. Conceptual Density Functional Theory (CDFT) and Computational Peptidology (CP) are computational approaches used to study the behavior of atoms, molecules, and peptides. In this study, we present the results of our investigation of the chemical reactivity and ADMET properties of Stellatolides A-H using a novel computational approach called Conceptual DFT-based Computational Peptidology (CDFT-CP). Our study uses CDFT and CP to predict the reactivity and stability of molecules and to understand the behavior of peptides at the molecular level. We also predict the ADMET properties of the Stellatolides A–H to provide insight into their effectiveness, potential side effects, and optimal dosage and route of administration, as well as their biological targets. This study sheds light on the potential of Stellatolides A–H as promising candidates for drug development and highlights the potential of CDFT-CP for the study of other natural compounds and peptides.

## 1. Introduction

Stellatolides are a group of natural compounds that have been isolated from a variety of marine organisms, including sponges and tunicates. They are characterized by their distinctive chemical structure, which features a complex macrolide ring system with multiple functional groups. Stellatolides have generated significant interest in the pharmaceutical industry due to their potential as a source of novel drugs for the treatment of various diseases [1,2,3,4,5,6].

The chemistry of stellatolides is complex and fascinating. These compounds are composed of a large macrolide ring system, which is formed through a series of complex biosynthetic pathways. The exact mechanisms by which stellatolides are synthesized in nature are not yet fully understood, but it is believed that they are produced by a combination of enzymatic and non-enzymatic processes [1,2,3,4,5,6].

Stellatolides have been shown to have a range of biological activities, including antitumor, antimicrobial, and anti-inflammatory properties. These activities are believed to be mediated by the ability of stellatolides to interact with specific biological targets in the body, such as enzymes or receptors [1,2,3,4,5,6].

One of the most exciting aspects of stellatolides is their potential as pharmacological drugs. Researchers have identified several stellatolide derivatives that show promising activity against a range of diseases, including cancer and infectious diseases. These compounds have been shown to have potent anticancer activity against a range of tumor cell lines, and they may offer a promising alternative to traditional chemotherapy drugs [7,8,9,10].

Stellatolides also have potential as a source of new antibiotics. With the rise of antibiotic-resistant bacteria, there is a growing need for new drugs that can effectively treat infections [11]. Stellatolides have been shown to have potent antimicrobial activity against a range of bacteria, including methicillin-resistant Staphylococcus aureus (MRSA) and vancomycin-resistant Enterococcus (VRE) [12].

In summary, stellatolides are a fascinating group of natural compounds with a wide range of biological activities and potential as pharmacological drugs. Further research is needed to fully understand their mechanisms of action and to develop effective drug candidates, but they represent a promising area of research in the pharmaceutical industry [1,2,3,4,5,6,7,8,9,10].

Although it has been asserted that peptides have no future as therapeutic drugs due to the violation of Lipinski’s rules [13] related to their size, solubility, and flexibility, leading to poor bioavailability, it should be mentioned that this assertion does not hold for cyclopeptides [14]. There is much published research validating this phenomena and also explaining the mechanisms for the drug delivery into the body [15,16,17,18,19,20,21]. Moreover, there are ongoing studies on the use of different substances as nanocarriers allowing the correct delivery of potential therapeutic peptides [22,23,24,25].

Chemical Reactivity Theory (CRT) is a branch of chemistry that seeks to explain and predict the behavior of chemical reactions based on the electronic structure of molecules. The basic idea behind CRT is that the reactivity of a molecule depends on its electronic structure, specifically the distribution and arrangement of electrons in the molecule. This electronic structure determines how the molecule interacts with other molecules or ions, leading to chemical reactions [26]. One of the key concepts in CRT is the concept of frontier molecular orbitals. These are the highest occupied molecular orbital (HOMO) and the lowest unoccupied molecular orbital (LUMO) of a molecule. The HOMO represents the electron density that is most likely to participate in chemical reactions, while the LUMO represents the electron density that is most easily available to accept electrons [27].

One of the most successful approaches to CRT is the so-called Conceptual Density Functional Theory (CDFT), which is a theoretical framework used to study the behavior of atoms and molecules in chemistry and physics. The conceptual aspect of DFT refers to the interpretation of the electronic density in terms of chemical concepts, such as bond formation and reactivity. For example, electron density can be used to predict chemical reactions, identify reactive sites, and explain the properties of materials. Thus, by interpreting electron density in terms of chemical concepts, CDFT provides insight into the behavior of molecules and materials and can be used to predict and understand chemical reactions and material properties [27,28,29,30,31,32,33,34].

Computational Peptidology (CP) is a field of research that uses computational methods to study the properties and behavior of peptides, which are small chains of amino acids that are important in many biological processes. The basic idea behind CP is to use computer simulations and modeling to understand the behavior of peptides at the molecular level. This involves studying the interactions between individual amino acids, as well as the interactions between peptides and other molecules in their environment, such as proteins or membranes. By considering both approaches, CDFT and CP, for the study of peptides, we have developed Conceptual DFT-based Computational Peptidology (CDFT-CP), which is a computational approach that uses DFT, CDFT, and CP to study the properties and behavior of peptides at the molecular level. CDFT-CP uses concepts such as electronegativity, hardness, softness, and electrophilicity to predict the reactivity and stability of molecules [35,36,37,38,39,40,41,42].

The objective of this research is to report the results of a CDFT-CP study of the chemical reactivity properties of the Stellatolides A–H marine cyclopeptides [7,9]. This will be complemented by the computational prediction of their ADMET properties [43] and biological targets. ADMET stands for Absorption, Distribution, Metabolism, Excretion, and Toxicity and refers to a set of processes that a drug undergoes in the body [44,45]. Comprehending the ADMET characteristics of a pharmaceutical compound holds significance in the realm of drug development. This understanding aids in foreseeing the drug’s efficacy, potential adverse reactions, and the most suitable dosage and method of delivery [46]. Furthermore, in preparation for a forthcoming molecular docking study aimed at elucidating the interactions between the analyzed cyclopeptides and their respective biological targets, we will employ computational pharmacokinetics software to predict these interactions, relying on their simplified molecular-input line-entry system (SMILES) descriptions.

## 2. Results and Discussion

**Figure 1:**
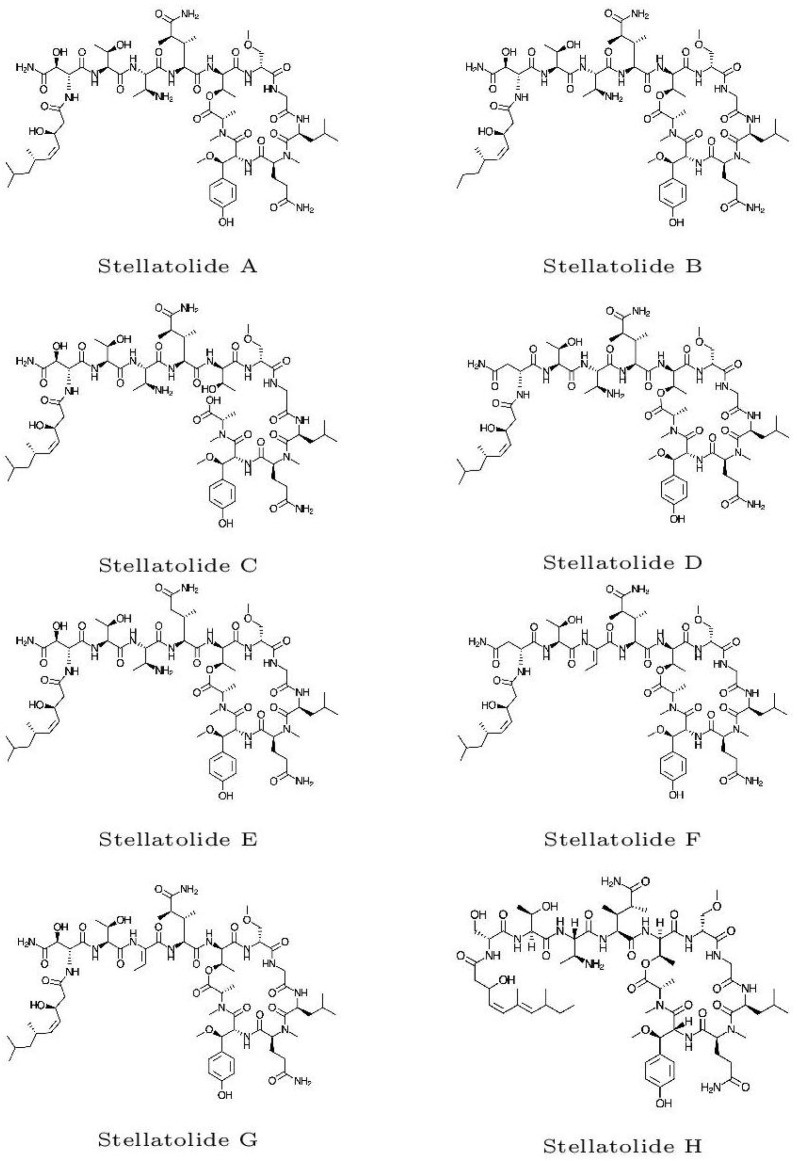
Graphical sketches of the initial molecular structures of the Stellatolides A–H.

The initial molecular configurations of the antimicrobial marine cyclopeptides slated for examination were acquired from ChemSpider (https://www.chemspider.com, accessed on 15 April 2023). ChemSpider is a no-cost chemical structure repository that offers rapid text and structural search capabilities for more than 100 million structures sourced from numerous data outlets. It also encompasses details about physical, chemical, and biological attributes, interactive spectra, and references to scholarly works. These initial molecular structures are shown in Figure 1.

It is often assumed that the quality of a density functional can be assessed by comparing its results to experimental values or to the results of post–Hartree–Fock calculations. However, this is not always possible, as experimental data may not be available for the systems being studied, or the molecules may be too large for accurate calculations to be feasible.

To address this issue, a protocol called KID (Koopmans in DFT) has been developed [35,36,37,38,39]. This protocol aims to validate a density functional by assessing its internal consistency. The KID protocol has been previously shown to be effective for peptides, but it is worth further validating for the molecules considered in this study.

The KID protocol was implemented using the in-house-developed CDFT software tool. The results of this analysis are shown in Table 1:

The J${}_{I}$ and J${}_{A}$ descriptors consider differences between the HOMO and ionization energy (I), and between the LUMO and electron affinity (A), respectively. According to the Ionization Energy Theorem, A can be predicted by the SOMO, which is equivalent to the anion’s HOMO. To align with Koopmans’ theorem, we introduced the ΔSL descriptor, comparing SOMO and LUMO values. The J${}_{HL}$ descriptor verifies the difference between the H-L gap and the I-A gap calculated from energy differences of neutral, positive, and negative species.

The results in Table 1 show that the KID descriptor values are nearly 0, indicating that the MN12SX/Def2TZVP/H2O model chemistry is effective for verifying the Janak and Ionization Energy theorems. Although further calculations involve energy differences, accurate predictions for HOMO, LUMO, and H-L gap provide confidence in our estimations of chemical reactivity properties. These estimations are theoretically grounded, not accidental as often seen with other density functionals.

**Figure 2:**
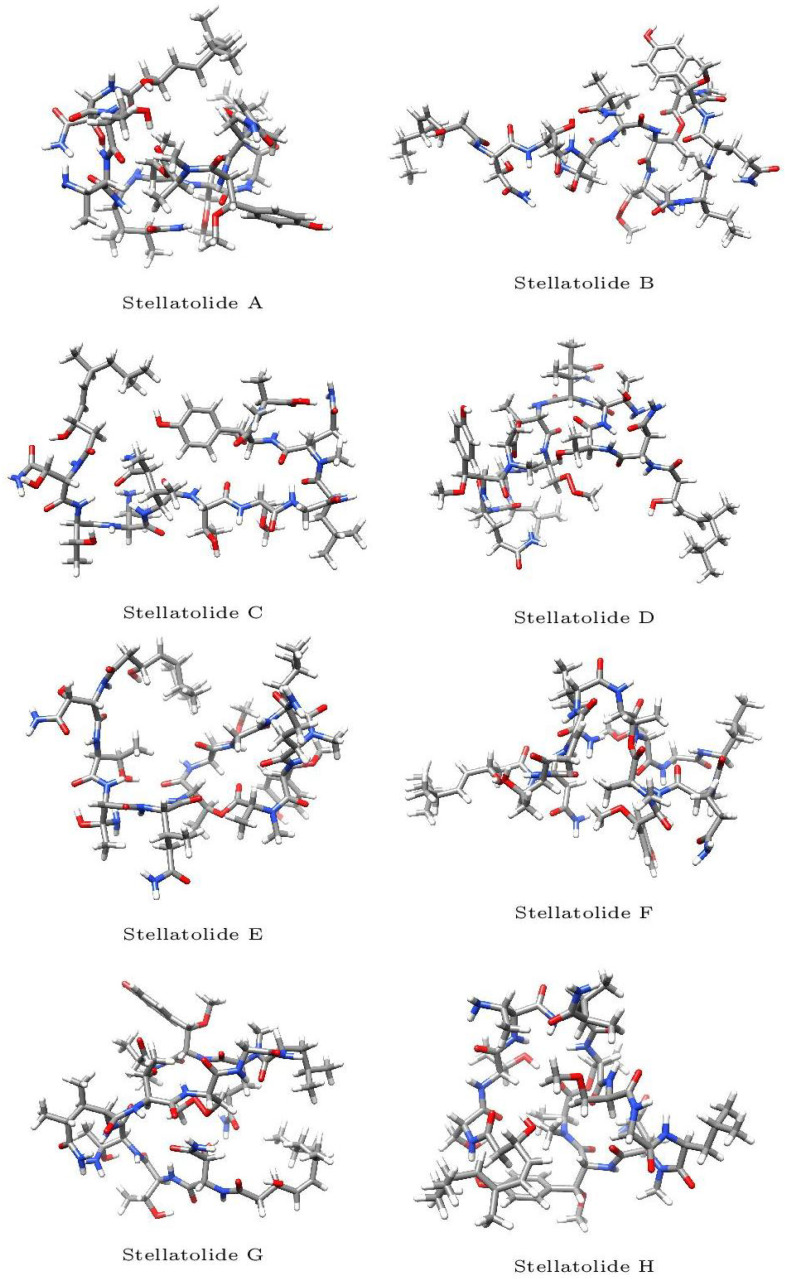
Optimized molecular structures of the Stellatolides A–H.

The optimized molecular structures of the eight members of the Stellatolides A–H family of cyclopeptides are displayed in Figure 2. These optimized structures have been obtained by following the procedure described in Section 3.1 of the Materials and Methods section.

The usual way to analyze the chemical reactivity of molecular systems through the consideration of Conceptual DFT is by reporting the values of the global and local descriptors that arise from this theory [27,28,29,30,31,32,33,34]. The three basic descriptors are the electronegativity χ, the global hardness η, and the global electrophilicity ω. The first is a measure of the tendency of an atom to attract electrons. It is a fundamental property of atoms that affects their chemical bonding and reactivity. In CDFT, electronegativity is defined as the Lagrange multiplier associated with the constraint that the total electronic chemical potential of a system is equal to a constant. This means that the electronegativity ω is a measure of the resistance of an atom to losing electrons, and it can be estimated through the operational formula $\chi =-\frac{1}{2}\left(I+A\right)\approx \frac{1}{2}\left(\epsilon_{L}+\epsilon_{H}\right)$ [27,28,29,30,31,32,33,34].

Global hardness η is a crucial concept within the realm of CDFT, which measures the resistance of a system to changes in its electron density when subjected to external perturbations. The global hardness can be used to predict the reactivity of a molecule. A molecule with a high global hardness η is less reactive than a molecule with a low global hardness. This is because a molecule with a high global hardness η is more difficult to polarize, and therefore less likely to react with other molecules. The formula $\eta =\left(I-A\right)\approx \left(\epsilon_{L}-\epsilon_{H}\right)$ is used for the evaluation of η, either considering energy differences or frontier orbitals [27,28,29,30,31,32,33,34].

The third basic descriptor, global electrophilicity ω [47], is particularly useful in predicting reactions and interactions between molecules. It can be expressed as a relation between χ and η as ω = $\mu^{2}/2\eta ={\left(I+A\right)}^{2}/4(I-A)\approx {\left(\epsilon_{L}+\epsilon_{H}\right)}^{2}/4(\epsilon_{L}-\epsilon_{H})$. A more electrophilic species tends to react with more nucleophilic ones, as electron flow will be from the nucleophile to the electrophile. This concept aids in understanding reaction mechanisms, molecular binding, and site-selectivity in chemical processes [27,28,29,30,31,32,33,34].

In addition to the global reactivity descriptors originating from Conceptual Density Functional Theory (DFT), Domingo and their colleagues [48,49,50,51,52] introduced a Nucleophilicity Index labeled as N. This index is established by evaluating the HOMO energy using the KS approach, wherein the reference point is shifted arbitrarily, centered around the tetracyanoethylene (TCE) molecule.

More-recently developed global reactivity descriptors are the electrodonating and electroaccepting powers: $\omega^{-}$ = ${\left(3I+A\right)}^{2}/16(I-A)\approx {\left(3\epsilon_{H}+\epsilon_{L}\right)}^{2}/16\eta$, $\omega^{+}$ = ${\left(I+3A\right)}^{2}/$$16(I-A)\approx {\left(\epsilon_{H}+3\epsilon_{L}\right)}^{2}/16\eta$, respectively, [29], and the net electrophilicity $\Delta \omega^{\pm }=\omega^{+}-\left(-\omega^{-}\right)=\omega^{+}+\omega^{-}$ [30], a comparison of the former two.

By utilizing the above-mentioned CDFT software tool as well as the MultiWFN package [53,54] applied to the results of the calculation of the electronic properties of the studied cyclopeptides, the values of the defined global reactivity descriptors could be obtained and they are displayed in Table 2:

From Table 2, the values of the electronegativity χ range from 3.2610 eV for Stellatolide A to 3.9451 eV for Stellatolide F. A slightly lower value is obtained for Stellatolide G, while the other considered peptides have similar intermediate values for this descriptor. If we now turn our attention to global hardness η, then it can be appreciated that the lowest value of this property is for the case of Stellatolide F, with 4.6145 eV. The results from both reactivity descriptors agree in that Stellatolide F will be the most reactive cyclopeptide of this family. The other members display larger values of η, meaning that they will be less reactive than the H species. Notwithstanding, there are differences between Stellatolides F and G, which will be slightly more reactive than Stellatolides A-E. As mentioned earlier, the global electrophilicity ω represents a balance between χ and η. The results for this descriptor from Table 2 range between 1.1759 eV for Stellatolide C to 1.6864 eV for Stellatolide F. Stellatolides A-E together with Stellatolide H display the lowest values of ω, while Stellatolide G presents a bit greater number. The analysis of the first three basic chemical reactivity descriptors lead us to the conclusion that Stellatolide F will the most reactive of all the cyclopeptides considered in this research.

As mentioned earlier, the nucleophilicity index N is another important quantity that serves as a descriptor of chemical reactivity. From Table 2, values of N range from 2.8066 eV for Stellatolide C to 3.1565 eV for Stellatolide H. As a matter of fact, all the members of this family have similar values for the nucleophilicity index N, with the exception of stellatolide H, which is a little larger. According to the classification proposed by Domingo et al. [48,49,50,51,52], while Stellatolide H may be regarded as a strong nucleophile, the others can be considered as moderate ones.

The last three descriptors in Table 2 are interrelated. As is usual for this kind of molecule, the electrodonating power $\omega^{-}$ is large that the corresponding electroaccepting power $\omega^{+}$. This behavior could be attributed to the cyclic structure of the peptides and their large number of conjugated bonds, as well as the presence of several O and N atoms. Stellatolide F possesses the larges value of $\omega^{-}$, followed by Stellatolide G, while the others display almost the same values for this chemical reactivity descriptor. For the case of the electroaccepting power $\omega^{+}$, again Stellatolide F is the most important among the whole family of cyclopeptides, with significant differences to the other molecules, with the exception of Stellatolide G. As expected, the net electrophilicity $\Delta \omega^{\pm }$, being defined as a way to compare $\omega^{-}$ with $\omega^{+}$, shows the same behavior for the cyclopeptides as for the other two reactivity descriptors, allowing the conclusion that Stellatolide F will be the most reactive cyclopeptide of all those considered in this work.

The chemical reactivity descriptors that have been reported are classified as global indicators as they play a crucial role in predicting the overall properties of molecules. Nevertheless, there are instances where delving into the characteristics of specific regions within molecular systems becomes intriguing. By recognizing that distinct chemical reactivity is confined to various zones within these molecules, we gain insights into their behavior. This prompts the use of what are known as local reactivity descriptors, which stem from the principles of Conceptual DFT (CDFT) [27,28,29,30,31,32,33,34].

The Fukui functions (FF), cited in references such as [27,28,29,30,31,32,33,34], stand out as the most prevalent local descriptors in Conceptual Density Functional Theory (CDFT). These functions can be categorized into nucleophilic, electrophilic, and radical Fukui functions, their classification contingent on the incoming reagent and the specific attack site. This classification enables FF to meticulously characterize individual atoms within a molecule where reactions might take place.

While this approach has proven incredibly valuable and achieved significant success in the context of small molecules, its application to larger molecular systems poses challenges. This is due to the tendency for the resulting values to become exceedingly minute, making it arduous to differentiate between them.

In CDFT, the dual descriptor, often abbreviated as DD, stands as a powerful tool utilized for the examination of molecule reactivity [55,56]. This descriptor furnishes a quantification of a molecule’s nucleophilic and electrophilic traits, thus establishing its worth as a predictive instrument in anticipating chemical reactivity.

The derivation of the dual descriptor DD emanates from the frontier molecular orbitals, namely, the HOMO and LUMO of the molecule. Via the HOMO and LUMO, one can obtain the corresponding electrophilic and nucleophilic Fukui functions, which in turn allow for the computation of the dual descriptor DD [55,56,57,58].

The dual descriptor DD boasts an array of chemistry applications, encompassing catalyst design, chemical reaction prognosis, and chemical reactivity analysis. This tool serves as a valuable asset for comprehending the electronic structure of molecules, enabling the prediction of their conduct in chemical reactions.

By harnessing the dual descriptor DD, one can foresee a molecule’s reactivity toward nucleophilic or electrophilic assaults. A markedly positive value of this descriptor signifies the molecule’s proficiency as an electrophile, while a notably negative value suggests its competence as a nucleophile [55,56,57,58].

**Figure 3:**
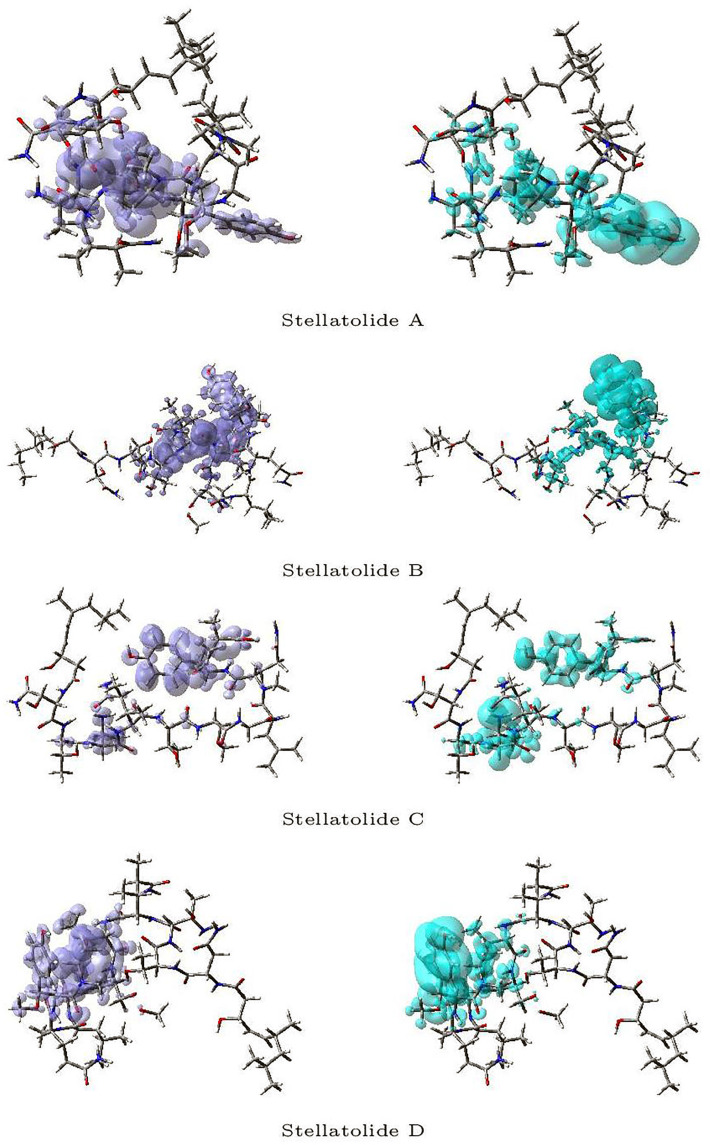
Graphical representation of the dual descriptor DD of the Stellatolides A–D. **Left**: DD > 0, **Right**: DD < 0.

**Figure 4:**
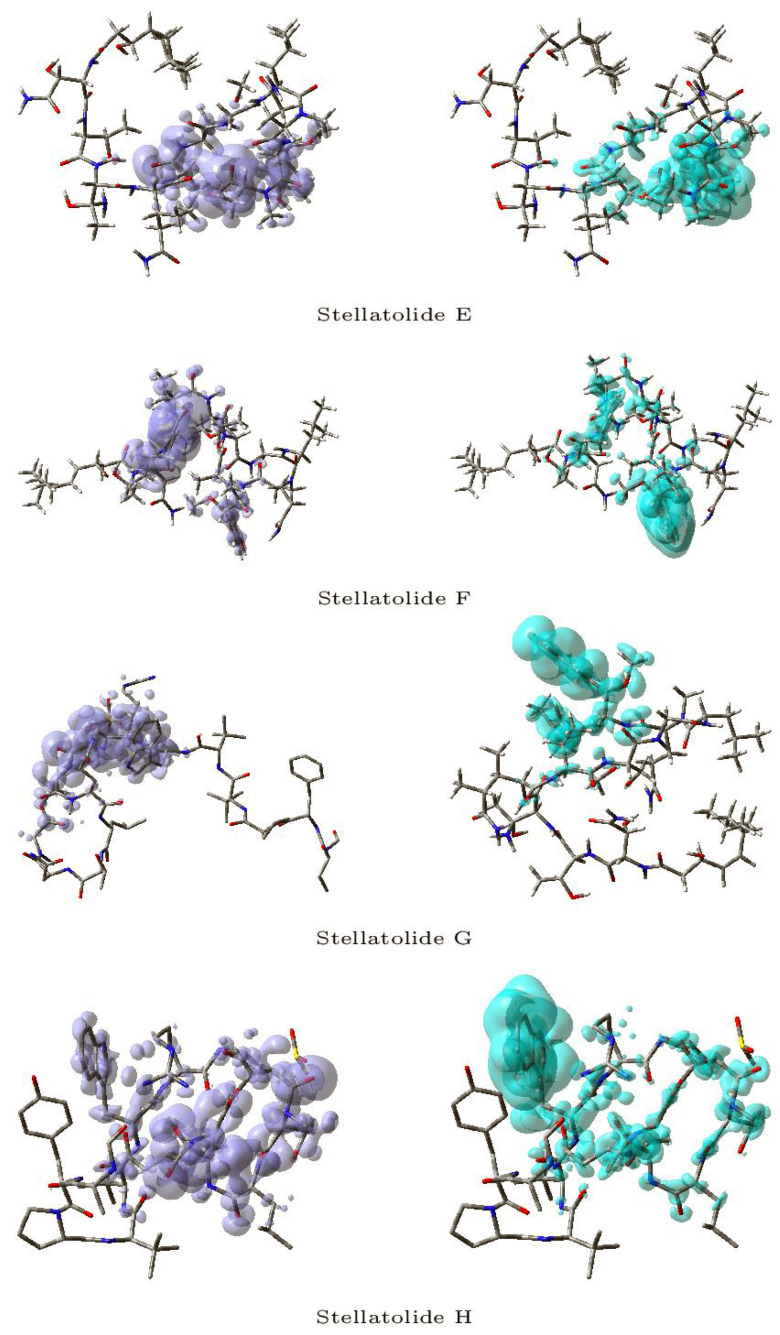
Graphical representation of the dual descriptor DD of the Stellatolides E–H. **Left**: DD > 0, **Right**: DD < 0.

Figure 3 and Figure 4 display a graphical representation of the dual descriptor DD of the Stellatolides A–H, with the images on the left indicating the zones where DD > 0, while the images on the right indicating the areas where DD < 0.

Upon reviewing Figure 3 and Figure 4, it becomes evident that it is indeed possible to discern specific regions within the peptides that are prone to susceptibility towards either nucleophilic or electrophilic attacks. This capability proves to be immensely valuable, as it lays the groundwork for the prospective evaluation of these molecular systems as potential therapeutic agents.

Online platforms for open bioactivity prediction, such as Molinspiration by Molinspiration Cheminformatics in Slovensky Grob, Slovak Republic, have proven instrumental in assessing molecular properties and bioactivity scores. This is achieved through an analysis of the chemicals in question, utilizing the Simplified Molecular Input Line Entry Specification (SMILES) notation. Molinspiration serves as an adept system for calculating drug-likeness information, while SwissTargetPrediction also contributes to this endeavor [59]. Notably, SwissTargetPrediction aids in predicting various targets, including g-protein coupled receptors (GPCR) ligands, ion channel modulators, kinase inhibitors, nuclear receptor ligands, protease inhibitors, and other enzyme targets (http://www.molinspiration.com/cgi-bin/properties, accessed on 15 April 2023).

In line with the information garnered from a CDFT analysis to gauge the chemical reactivity of the studied marine cyclopeptides, Table 3 furnishes bioactivity scores for these peptides across a spectrum of targets. These targets encompass GPCR ligands, ion channel modulators, nuclear receptor ligands, kinase inhibitors, protease inhibitors, and enzyme inhibitors. The scores play a pivotal role in predicting the peptides’ corresponding biological targets, an indispensable facet of drug development that offers profound insights into the potential therapeutic applications of these compounds [46].

The bioactivity scores associated with the analyzed cyclopeptides can be comprehended in the following manner: considered active when the bioactivity score exceeds 0, categorized as moderately active if the score falls within the range of −5.0 to 0.0, and deemed inactive when the bioactivity score is below −5.0. This categorization system allows for a clear interpretation of the cyclopeptides’ bioactivity levels.

ADMET properties play a pivotal role in the realm of drug discovery. These properties collectively determine how a potential drug candidate interacts within the human body, influencing its efficacy and safety profile [43,44,46].

Computational prediction of ADMET properties has emerged as an indispensable tool in the drug discovery process. Traditional experimental methods for assessing these properties are time-consuming, costly, and often carried out in the later stages of drug development. In contrast, computational models utilize data-driven algorithms to swiftly evaluate a compound’s ADMET characteristics, even in the early stages of drug design.

Utilizing computational approaches, researchers can swiftly analyze extensive chemical libraries to pinpoint promising candidates exhibiting favorable ADMET profiles. This expedites the drug discovery timeline and diminishes the chances of allocating resources to compounds that may face significant obstacles during clinical trials due to unfavorable ADMET properties.

Furthermore, computational prediction assists in identifying potential toxicity issues, enabling researchers to eliminate compounds with a higher risk of adverse effects. This proactive strategy not only minimizes the chances of late-stage failures but also enhances patient safety.

In summary, the computational prediction of ADMET properties revolutionizes the drug discovery landscape by providing timely insights into a compound’s behavior within the body. This predictive power not only expedites the identification of viable drug candidates but also contributes to more efficient, cost-effective, and safer drug development processes.

The ADMET parameters for the eight members of the Stellatolides A–H family estimated by utilizing SwissADME [60] and pkCSM [61] software are displayed in Table 4, Table 5, Table 6, Table 7 and Table 8:

From Table 4, it can be seen that the values of the excretion parameters are the same for the eight cyclopeptides with the exception of Caco-2 Permeability.

The Caco-2 cell line plays a pivotal role in the field of ADMET within the realm of drug development. Caco-2 cells are derived from human colon carcinoma and are widely used as an in vitro model to simulate the behavior of the intestinal epithelium, specifically for drug absorption studies [43,44,46]. These cells form a monolayer that mimics the intestinal lining, allowing researchers to assess how well a drug can be absorbed through the gastrointestinal tract. This information is crucial because a drug’s bioavailability, or the extent to which it reaches systemic circulation, heavily depends on its ability to be absorbed by the intestines. In drug development, Caco-2 assays are employed to predict a drug’s potential for oral absorption thus allowing the estimation of the likelihood of a drug being absorbed efficiently or encountering obstacles due to efflux transporters. This information guides medicinal chemists and pharmaceutical scientists in optimizing drug structures to enhance their bioavailability. In this regards, Stellatolides C, D, and G excel over the others, while Stellatolide H presents a very different small value.

Distribution is a critical aspect of ADMET. Once a drug is absorbed into the bloodstream, it needs to be effectively distributed to its target tissues and organs to exert its therapeutic effects. Understanding a drug’s distribution properties is essential for optimizing its efficacy and minimizing potential side effects. Distribution can also be influenced by factors like tissue perfusion rates and the presence of drug transporters. Tissues with high blood flow receive more drug, while those with low blood flow receive less [43,44,46].

Volume of Distribution at Steady State (VDss) is a pharmacokinetic parameter used in the field of clinical pharmacology to describe the theoretical volume into which a drug would need to distribute in order to account for its total amount in the body at steady state concentration. VDss provides valuable insights into a drug’s distribution characteristics within the human body. The concept of VDss is particularly useful for understanding how extensively a drug distributes beyond the bloodstream into various tissues and compartments. It helps determine whether a drug tends to stay predominantly within the blood plasma or if it has a tendency to accumulate in specific tissues or organs. VDss is a crucial parameter in drug dosing calculations. It aids in determining the appropriate dosage needed to achieve the desired therapeutic effect based on the drug’s distribution characteristics. Drugs with high VDss may require larger loading doses to rapidly establish therapeutic levels, while drugs with low VDss may need lower loading doses due to their tendency to remain in the bloodstream [44].

Fraction unbound, also known as the free fraction or unbound fraction, is a crucial concept in the field of ADMET within drug development. It refers to the proportion of a drug that exists in its pharmacologically active, unbound form within the bloodstream. In pharmacology, only the unbound fraction of a drug is capable of interacting with its target receptors and exerting therapeutic effects. The fraction unbound is particularly relevant in understanding a drug’s distribution, as only the unbound drug can pass through cell membranes and diffuse into tissues to reach its target site.

Blood–Brain Barrier (BBB) permeability is a fundamental concept in neuroscience and pharmacology, playing a critical role in understanding how substances, including drugs, interact with the central nervous system (CNS). The BBB is a specialized barrier that separates the bloodstream from the brain and spinal cord, tightly regulating the passage of molecules between these two compartments. Understanding BBB permeability is of utmost importance in drug development, especially for drugs targeting neurological disorders. A drug’s ability to cross the BBB is critical for it to exert therapeutic effects on the brain. Some drugs are designed to have high BBB permeability, while others are engineered to stay outside the CNS to prevent unwanted side effects.

Central Nervous System (CNS) permeability refers to the ability of substances, particularly drugs, to cross the blood–brain barrier (BBB) and gain access to the brain and spinal cord. The CNS is a highly protected and vital area of the body, and its permeability properties significantly influence the efficacy of drugs targeting neurological disorders. CNS permeability is a pivotal consideration in drug development for neurological disorders. Understanding the factors influencing CNS permeability and designing drugs that can effectively cross the blood–brain barrier are essential for developing treatments that target the central nervous system with precision and efficacy.

In summary, distribution properties play a vital role in determining a drug’s effectiveness and potential side effects. By studying how a drug is distributed within the body, researchers can optimize drug formulations, dosing regimens, and delivery strategies to ensure that the right concentration reaches the intended target while minimizing the risk of adverse effects in non-target tissues. Thus, the results from Table 5 may be of great significance when developing therapeutic drugs through the consideration of Stellatolides A–H. It can be appreciated that Stellatolide C will display the largest VDss value, which will be the lowest for Stellatolide F. The fraction unbound is similar for all the compounds, with the exception of Stellatolides C and H. The BBB permeability will also be similar for all the peptides, except for Stellatolide C, for which it is slightly lower. Finally, for CNS permeability, Stellatolide C displays the largest value, and Stellatolide F the lowest one, while Stellatolides A, B, and E have intermediate values.

Metabolism properties are a crucial aspect of ADMET studies in the field of drug development [43,44,46]. Metabolism refers to the biochemical processes that transform a drug into different compounds, often resulting in its inactivation and elimination from the body. Understanding a drug’s metabolism properties is essential for optimizing its efficacy, safety, and dosing regimens. Drug metabolism primarily occurs in the liver, where enzymes break down drugs into metabolites that are more water-soluble and can be excreted through urine or bile. From Table 6, it can be appreciated that all the studied cyclopeptides will display a negative behavior toward being either substrate or inhibitor of different cytochrome enzymes.

Excretion is one of the key properties within ADMET that focuses on the elimination of drugs and their metabolites from the body. This process primarily occurs through the kidneys via urine, but can also involve other routes like bile, sweat, and exhaled air. The excretion property of ADMET plays a pivotal role in determining a drug’s dosing regimen and potential toxicity. Efficient excretion helps maintain appropriate drug levels in the body, preventing accumulation that could lead to adverse effects. Conversely, impaired excretion can lead to drug accumulation, prolonged therapeutic effects, or even harmful side effects [43,44,46].

Clearance is a fundamental pharmacokinetic parameter as it directly influences a drug’s dosing regimen. It helps determine how often a drug needs to be administered to maintain therapeutic levels in the body and avoid toxicity due to drug accumulation. Total clearance takes into account two primary processes: renal clearance and hepatic clearance. Renal clearance refers to the removal of a drug via the kidneys, primarily through urine, while, hepatic clearance involves the metabolism and subsequent elimination of a drug by the liver. These two processes are often the dominant contributors to a drug’s total clearance.

Renal excretion is especially crucial, as the kidneys filter the bloodstream to eliminate drug molecules. This filtration process is influenced by factors such as the drug’s size, charge, and lipid solubility. Active transporters in the kidneys can also affect excretion, leading to variations in drug clearance among individuals. Renal OCT2 (Organic Cation Transporter 2) substrate refers to a drug or compound that can be transported into and out of cells in the kidneys by the OCT2 transporter protein. OCT2 is a type of transporter that plays a crucial role in the renal excretion of various organic cations, which are positively charged molecules.

It can be seen from Table 7, that all the cyclopeptides present a negative behavior for being a Renal OC2 substrate. Conversely, they display different activities under the Total Clearance test, with Stellatolides F and G showing positive values while all the others show negative values.

Toxicity is a significant aspect of the ADMET framework that focuses on assessing the potential harmful effects of a drug or compound on living organisms [43,44,46]. It plays a critical role in drug development, as understanding and mitigating potential toxicities is essential for ensuring the safety and efficacy of pharmaceutical products. Toxicity can manifest in various ways, including adverse effects on organs, tissues, cells, and biochemical processes. There are different types of toxicity, such as acute toxicity (rapid and severe effects shortly after exposure) and chronic toxicity (long-term and cumulative effects). The toxic effects can be dose-dependent, where higher doses lead to more severe reactions, or idiosyncratic, occurring unpredictably in certain individuals due to genetic variations. In summary, toxicity assessment within the ADMET framework is crucial for identifying and managing potential harmful effects of drugs and compounds. It guides drug development by ensuring the safety of pharmaceutical products and contributing to better patient outcomes. Thus, we believe that the results from Table 8 will be of great help in predicting the possible toxicities of the Stellatolides A–H to guide future developments of therapeutic drugs based on these marine cyclopeptides. It can be observed that the studied peptides will not exhibit AMES toxicity and will not act as hERG I inhibitors. Among them, Stellatolide H is projected to have the highest MTD (Human), while Stellatolides F and G display the lowest values, with the others falling in between. All the studied peptides will function as hERG II inhibitors, except for Stellatolide C. The ORAT (LD50) is nearly identical for all the compounds, and the same can be said for the ORCT (LOAEL), except in the case of Stellatolide H. All the molecules yield a positive result for hepatotoxicity and a negative result for skin sensitization. In terms of toxicity, the numbers for *T. Pyriformis* are consistent across all the compounds, while the Minnow values will be similar between them, except for Stellatolide H, which has the highest value, and Stellatolide F, which has the lowest one.

Certainly, it is not feasible to establish a direct correlation between the ADMET parameter values showcased in this context and the earlier CDFT chemical reactivity results. Nevertheless, it is important to acknowledge that these two sets of findings can be viewed as providing complementary information. To elaborate further, while the ADMET parameters offer insights into various aspects of a compound’s pharmacokinetics and safety profile, the CDFT chemical reactivity results shed light on its chemical behavior and reactivity patterns. Consequently, while they may not directly align, the amalgamation of these distinct datasets enriches our overall understanding of the compound’s properties and potential applications.

**Figure 5:**
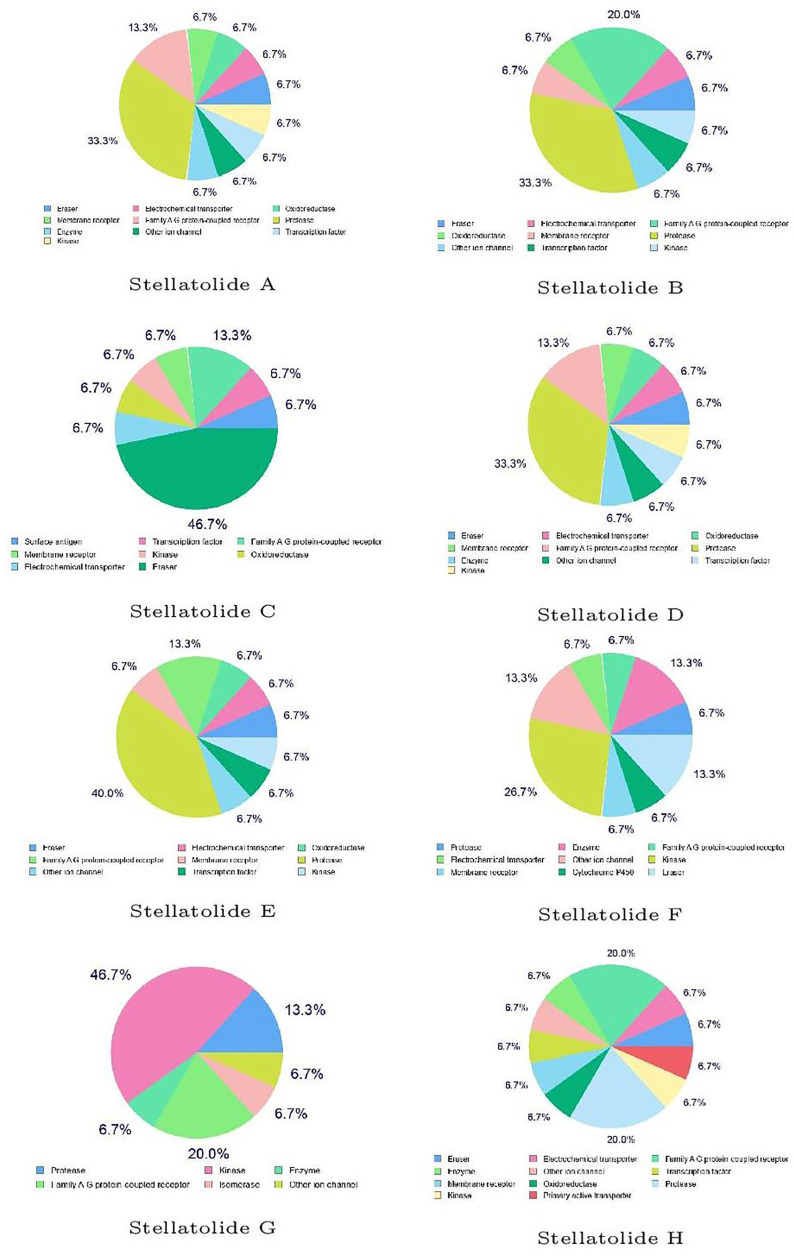
Predicted biological targets of the Stellatolides A–H cyclopeptides.

Finally, the predicted biological targets of the Stellatolides A–H cyclopeptides obtained by utilizing SwissTargetPrediction software are shown in Figure 5:

The results presented in Figure 5 serve as examples of the complementarity between the various predictions made in this study, even though they cannot be directly correlated. While each peptide may have the potential to interact with different biological targets, the peptide with the highest percentage is likely the most probable. Therefore, it is evident that some of these peptides might interact with proteases, while others could engage with kinases, and Stellatolide H may have an affinity for GPCRs. Although it is not feasible to draw direct comparisons between the peptides studied here and the results obtained for other peptide families, we believe that the findings we have obtained will prove valuable for an upcoming molecular docking study. This future study aims to elucidate the interactions between the analyzed cyclopeptides and their respective biological targets.

## 3. Materials and Methods

### 3.1. Density Functional Theory (DFT) Calculations

The Kohn–Sham (KS) methodology involves assessing the energy and density of a specific molecular system, along with the orbital energies associated with the frontier orbitals such as the Highest Occupied Molecular Orbital (HOMO) and Lowest Unoccupied Molecular Orbital (LUMO) [62,63,64,65]. This approach is particularly useful for evaluating quantitative characteristics associated with Conceptual DFT descriptors [27,28,29,30,31,32,33,34].

In the present investigation, the identification of the molecular conformers was carried out utilizing MarvinView 17.15, a software package provided by ChemAxon (http://www.chemaxon.com, accessed on 15 April 2023). Molecular Mechanics calculations were performed using the MMFF94 force field [66,67,68,69,70]. Subsequently, a geometry optimization and frequency calculation were conducted using the Density Functional Tight Binding (DFTBA) approach [71]. The purpose of this final stage was to ensure the stability of the optimized structures by confirming the absence of imaginary frequencies, which serves as a criterion for determining their status as energy minima in the overall energy landscape.

The electronic properties and chemical reactivity descriptors of the molecules under investigation were analyzed using the MN12SX/Def2TZVP/H2O model chemistry [72,73,74]. The molecular structures were previously optimized as mentioned earlier, and this particular model chemistry was selected because it allows for the validation of the ‘Koopmans in DFT’ (KID) procedure [36,37,38,39,40,41,42]. The calculations were performed using Gaussian 16 software [71] and the SMD solvation model [75]. The chosen model chemistry comprises the MN12SX screened-exchange density functional [72] and the Def2TZVP basis set [73,74]. Throughout the analysis, the molecules were treated as neutral, while the radical anion and cation were considered in the doublet spin state.

### 3.2. In Silico Pharmacokinetics Analysis and ADMET Study

To gain insight into the potential therapeutic attributes of the peptides under investigation, we utilized the SMILES (Simplified Molecular Input Line Entry Specification) notation of each compound. This notation was obtained by utilizing ChemDoodle 11.3.0 software. Subsequently, we employed Molinspiration software from Molinspiration Cheminformatics (https://www.molinspiration.com, accessed on 15 April 2023) to compute the molecular properties associated with drug-like characteristics.

The objective of exploring similarity searches within the chemical realm of compounds, comparing them to the compounds under investigation and known pharmacological properties, was successfully achieved by utilizing online Molinspiration software. This software package facilitated the prediction of bioactivity scores for various drug targets, including GPCR ligands, kinase inhibitors, ion channel modulators, enzymes, and nuclear receptors.

In order to assess the potential bioactivity of the antimicrobial marine cyclopeptides examined in this research, we employed a webtool called SwissTargetPrediction [59]. This tool efficiently predicts protein targets for small molecules. By utilizing this associated website, it was possible to estimate the most likely macromolecular targets of a small molecule assumed to be bioactive. The prediction process incorporates a combination of 2D and 3D similarity analysis using a comprehensive library of over 370,000 known active compounds targeting more than 3000 proteins from three distinct species.

During the process of developing a novel medication, it is crucial to understand the potential path of the therapeutic compound within the body, a concept referred to as pharmacokinetics. This involves assessing various aspects known as Absorption, Distribution, Metabolism, Excretion, and Toxicity (ADMET) parameters, which provide insights into the compound’s effects. Rather than relying solely on experimental methods, computer models can serve as an alternative approach to determine these parameters. In our study, we utilized SwissADME software, which is accessible online, to estimate a subset of ADME parameters [44,60].

Additional information about the Pharmacokinetics and ADMET properties were obtained by resorting to pkCSM [61], a software tool for the prediction of small-molecule pharmacokinetic properties using graph-based signatures or SMILES and that can be accessed through it associated webpage (https://biosig.unimelb.edu.au/pkcsm/, accessed on 15 April 2023). Chemicalize, which is software developed by ChemAxon (http://www.chemaxon.com, accessed on 15 April 2023), was used for name-to-structure generation and the prediction of several properties related to Chemoinformatics (http://chemicalize.com/) (accessed on 20 March 2023).

## 4. Conclusions

In conclusion, the investigation presented in this study offers a comprehensive understanding of the chemical reactivity, stability, and ADMET properties of Stellatolides A–H, utilizing the innovative computational approach known as Conceptual DFT-based Computational Peptidology (CDFT-CP). The promising biological activities exhibited by these Stellatolides, such as their antitumor, antimicrobial, and anti-inflammatory properties, underscore their potential as candidates for drug development.

The integration of Chemical Reactivity Theory (CRT), Conceptual Density Functional Theory (CDFT), and Computational Peptidology (CP) has provided valuable insights into the electronic structure and behavior of Stellatolides A–H. This approach not only advances our understanding of these natural compounds at the molecular level but also sets a precedent for the study of other natural compounds and peptides.

The predictions of ADMET properties based on the CDFT-CP approach offer significant implications for the potential therapeutic application of Stellatolides A–H. By elucidating their effectiveness, potential side effects, and appropriate dosage and administration routes, this study contributes to informed decision-making in drug development.

The outcomes of this research highlight the potential of Stellatolides A–H as promising drug candidates and emphasize the role of computational methodologies in accelerating the drug discovery process. The synergistic approach of combining various computational techniques enables a more holistic evaluation of these compounds, bridging the gap between theoretical insights and practical applications.

In conclusion, the findings presented in this study offer a glimpse into the future of drug development, where computational methods like CDFT-CP play a pivotal role in unveiling the potential of natural compounds and peptides. As the field of ADMET continues to evolve, studies like this pave the way for safer and more effective therapeutic interventions.

## Figures and Tables

**Table 1:** Predicted Frontier Orbital Energies, H-L Gap, and the KID Descriptors (all in eV) for the Stellatolides A–H.

Molecule	HOMO	LUMO	SOMO	H-L Gap	J${}_{I}$	J${}_{A}$	J${}_{\mathrm{HL}}$	ΔSL
Stellatolide A	−6.2605	−0.9396	−0.9192	5.3209	0.026	0.011	0.028	0.020
Stellatolide B	−6.2418	−1.0234	−1.0226	5.2183	0.035	0.001	0.035	0.001
Stellatolide C	−6.3147	−0.8531	−0.9241	5.4616	0.061	0.029	0.067	0.071
Stellatolide D	−6.2681	−1.1480	−1.1061	5.1201	0.034	0.015	0.037	0.042
Stellatolide E	−6.2118	−1.0876	−1.0787	5.1242	0.032	0.006	0.032	0.009
Stellatolide F	−6.2524	−1.6379	−1.6349	4.6145	0.026	0.001	0.026	0.003
Stellatolide G	−6.2722	−1.4128	−1.3690	4.8594	0.038	0.017	0.042	0.044
Stellatolide H	−5.9647	−1.1255	−1.1676	4.8393	0.027	0.019	0.033	0.042

**Table 2:** Global Reactivity Descriptors for the Stellatolides A–H: Electronegativity (χ), Global Hardness (η), Electrophilicity (ω), Nucleophilicity N, Electrodonating Power ($\omega^{-}$), Electroaccepting Power ($\omega^{+}$), and Net Electrophilicity ($\Delta \omega^{\pm }$) (all in eV).

Molecule	χ	η	ω	N	$\omega^{-}$	$\omega^{+}$	$\Delta \omega^{\pm }$
Stellatolide A	3.2610	5.3209	1.2179	2.8608	4.5684	0.9683	5.5366
Stellatolide B	3.6326	5.2183	1.2644	2.8795	4.6712	1.0386	5.7097
Stellatolide C	3.5839	5.4616	1.1759	2.8066	4.4850	0.9011	5.3861
Stellatolide D	3.7081	5.1201	1.3427	2.8531	4.8595	1.1515	6.0110
Stellatolide E	3.6497	5.1242	1.2998	2.9094	4.7447	1.0949	5.8396
Stellatolide F	3.9451	4.6145	1.6864	2.8688	5.6338	1.6887	7.3224
Stellatolide G	3.8425	4.8594	1.5192	2.8490	5.2634	1.4209	6.6843
Stellatolide H	3.5451	4.8393	1.2985	3.1565	4.6720	1.1269	5.7990

**Table 3:** Bioactivity Scores of the Stellatolides A–H Calculated on the Basis of the GPCR Ligand, Ion Channel Modulator, Nuclear Receptor Ligand, Kinase Inhibitor, Protease Inhibitor, and Enzyme Inhibitor Interactions.

Property	A	B	C	D	E	F	G	H
GPCR Ligand	−3.95	−3.95	−3.96	−3.95	−3.94	−3.95	−3.95	−3.94
Ion Channel Modulator	−4.01	−4.01	−4.01	−4.01	−4.00	−4.01	−4.02	−4.00
Nuclear Receptor Ligand	−4.03	−4.03	−4.03	−4.02	−4.02	−4.03	−4.03	−4.01
Kinase Inhibitor	−4.02	−4.02	−4.02	−4.01	−4.02	−4.02	−4.03	−4.00
Protease Inhibitor	−3.90	−3.90	−3.90	−3.89	−3.89	−3.89	−3.90	−3.89
Enzyme Inhibitor	−3.96	−3.96	−3.97	−3.96	−3.95	−3.96	−3.96	−3.95

**Table 4:** Absorption Properties of the Stellatolides A–H.

Property	A	B	C	D	E	F	G	H
Caco-2 Permeability	−0.760	−0.760	−0.893	−0.804	−0.648	−0.792	−0.810	−0.254
Intestinal Absorption (Human)	0	0	0	0	0	0	0	0
Skin Permeability	−2.735	−2.735	−2.735	−2.735	−2.735	−2.735	−2.735	−2.735
P-glycoprotein Substrate	Yes	Yes	Yes	Yes	Yes	Yes	Yes	Yes
P-glycoprotein I Inhibitor	No	No	No	No	No	No	No	No
P-glycoprotein II Inhibitor	No	No	No	No	No	No	No	No

**Table 5:** Distribution Properties of the Stellatolides A–H.

Property	A	B	C	D	E	F	G	H
VDss (Human)	−0.413	−0.413	−0.605	−0.338	−0.429	−0.191	−0.315	−0.361
Fraction Unbound (Human)	0.312	0.312	0.340	0.301	0.317	0.302	0.305	0.334
BBB Permeability	−3.081	−3.081	−3.661	−2.973	−3.076	−2.795	−3.053	−2.902
CNS Permeability	−7.266	−7.266	−7.945	−6.861	−7.300	−6.409	−6.835	−6.691

**Table 6:** Metabolism Properties of the Stellatolides A–H.

Property	A	B	C	D	E	F	G	H
CYP2D6 Substrate	No	No	No	No	No	No	No	No
CYP3A4 Substrate	No	No	No	No	No	No	No	No
CYP1A2 Inhibitor	No	No	No	No	No	No	No	No
CYP2C19 Inhibitor	No	No	No	No	No	No	No	No
CYP2C9 Inhibitor	No	No	No	No	No	No	No	No
CYP2D6 Inhibitor	No	No	No	No	No	No	No	No
CYP3A4 Inhibitor	No	No	No	No	No	No	No	No

**Table 7:** Excretion Properties of the Stellatolides A–H.

Property	A	B	C	D	E	F	G	H
Total Clearance	−1.731	−1.731	−0.965	−1.704	−1.694	0.611	0.623	−1.755
Renal OCT2 Substrate	No	No	No	No	No	No	No	No

**Table 8:** Toxicity Properties of the Stellatolides A–H.

Property	A	B	C	D	E	F	G	H
AMES Toxicity	No	No	No	No	No	No	No	No
Maximum Tolerated Dose (Human)	0.527	0.527	0.510	0.519	0.540	0.502	0.503	0.557
hERG I Inhibitor	No	No	No	No	No	No	No	No
hERG II Inhibitor	Yes	Yes	No	Yes	Yes	Yes	Yes	Yes
Oral Rat Acute Toxicity (LD50)	2.475	2.475	2.481	2.482	2.478	2.494	2.477	2.482
Oral Rat Chronic Toxicity (LOAEL)	4.837	4.837	4.473	4.354	5.070	4.616	4.673	5.887
Hepatotoxicity	Yes	Yes	Yes	Yes	Yes	Yes	Yes	Yes
Skin Sensitisation	No	No	No	No	No	No	No	No
*T. Pyriformis* Toxicity	0.285	0.285	0.285	0.285	0.285	0.285	0.285	0.285
Minnow Toxicity	18.264	18.264	18.517	17.368	17.894	16.506	17.155	19.686

## Data Availability

Data is contained within the article.
